# Uncrossing the ‘X’: Characterization of alternative alleles for *KSLX* in *Oryza*

**DOI:** 10.1016/j.phytochem.2025.114634

**Published:** 2025-08-08

**Authors:** Tristan Weers, Yiling Feng, Reuben J. Peters

**Affiliations:** Roy J. Carver Department of Biochemistry, Biophysics & Molecular Biology, Iowa State University, Ames, IA, 50011, USA

**Keywords:** Kaurene synthase-like, Labdane-related diterpenoids, Phytoalexins, Evolution

## Abstract

The widely cultivated Asian rice (*Oryza sativa*) produces a variety of physiologically relevant diterpenoid products, which range in effect from the phytohormone gibberellin, derived from *ent*-kaurene, to phytoalexins such as the momilactones, derived from *syn*-pimara-7,15-diene. Previous reports have shown functional variation in the kaurene synthase-like (KSL) genes responsible for synthesizing diterpene precursors to more specialized metabolites, leading to the creation of distinct diterpenoids from allelomorphic genes. Here is reported the product of two previously discovered but uncharacterized alleles of the unusual *KSLX*, representing a cross between (fusion of) the tandem pair *KSL8*-*KSL9p* found in most cultivars. The previously characterized allele (KSLXo) was reported to act on *syn*-copalyl pyrophosphate (*syn*-CPP) to produce *syn*-abieta-7,12-diene, precursor to the phytoalexin oryzalactone. However, at least one other functionally distinct allele was reported from the *O. sativa* pan-genome (KSLXn), along with another phylogenetically distinct allele found in *Oryza barthii* (KSLXb), but these were not further characterized. Here both KSLXn and KSLXb were found to selectively react with *syn*-CPP and produce *syn*-pimara-9(11),15-diene, a novel diterpene in rice. Additionally, evolution of this locus was investigated, with *KSLXb* hypothesized to be a functional *KSL9*. The striking complexity of this locus, which includes distinct composition (*KSL8-KSL9(p)* or *KSLX*) as well as allelomorphism of both *KSL8* and *KSLX*, suggests it is subject to balancing selection, consistent with the competing pressures exerted on phytoalexin biosynthesis. Regardless, the studies reported here clarify this additional example of allelomorphic variation in the rice KSL family, providing insight into the rice pan-genomic diterpenoid arsenal.

## 1. Introduction

The rice (*Oryza*) genus produces a wide variety of natural products to serve various roles in its growth, development, and defense. Many of these metabolites are diterpenoids, specifically labdane-related diterpenoids (LRDs), which serve various functions, most centrally as the gibberellin phytohormones, but also as more specialized phytoalexins and allelochemicals (Murphy and Zerbe, 2020; Valletta et al., 2023). The role of cultivated rice, particularly the Asian *O. sativa*, as a major staple crop makes these compounds and their biosynthesis of key agricultural interest.

LRDs are characterized by the bicyclization reaction catalyzed by (class II) diterpene cyclases with the general diterpenoid precursor, (*E,E, E*)-geranylgeranyl pyro-phosphate (Peters, 2025). Most commonly, this reaction produces the eponymous labdadienyl/copalyl pyro-phosphate (CPP), leading these enzymes to be designated CPP synthases (CPSs). All land plants must produce *ent-*CPP as well as contain a subsequently acting (class I) diterpene synthase for creation of *ent*-kaurene, designated kaurene synthase (KS), as required for gibberellin or related phytohormone biosynthesis (Wang et al., 2023). In cultivated Asian rice the relevant enzymes are OsCPS1 and OsKS1 (Sakamoto et al., 2004). As in many angiosperms, these have given rise to expanded gene families involved in more specialized metabolism. Reflecting this origin, members of the latter family are often termed KS-like, abbreviated KSLs (Zi et al., 2014).

In addition to *OsCPS1*, rice encodes another *ent*-CPP synthase, *OsCPS2*, which is associated with more specialized metabolism and co-expressed with many of the *KSLs* to initiate biosynthesis of a range of more specialized LRDs (Otomo et al., 2004; Prisic et al., 2004). Rice also encodes another inducible CPS, *OsCPS4*, which produces the stereo-chemically distinct *syn*-CPP (Otomo et al., 2004b; Xu et al., 2004), as well as *KSLs* specific for this stereoisomer, allowing for greater diversity in LRD biosynthesis (Lu et al., 2018). For example, OsKSL4 acts upon *syn*-CPP to yield the *syn*-pimara-7,15-diene precursor to the momilactones (Wilderman et al., 2004), allelochemicals shown to suppress growth of invasive weeds in rice-growing environments (Xu et al., 2012).

The KSLs initiate biosynthesis of various groups of LRDs derived from the resulting specific hydrocarbon skeleton – e.g., the *syn*-pimara-7,15-diene derived momilactones. Given the distinct activity exhibited by the rice KSLs, each family member then represents the capacity for another such group of more specialized LRDs. Intriguingly, several *KSLs* have been found to exhibit allelomorphism, with functionally distinct alleles found in the rice pan-genome (Zi et al., 2014). These have largely been assigned to the two predominant sub-species (ssp.) of Asian rice, ssp. indica or ssp. japonica, which are most closely related to the wild rice species *Oryza nivara* and *Oryza rufipogon*, respectively (Wing et al., 2018). The first example was discovered from competing studies of *OsKSL5*, which acts upon *ent*-CPP, but that from ssp. japonica (OsKSL5j) produces *ent*-pimaradiene (Kanno et al., 2006), while that from ssp. indica (OsKSL5i) produces *ent*-isokaurene (Xu et al., 2012). Similarly, although that from ssp. indica was originally termed *OsKSL11* due to phylogenetic divergence (Morrone et al., 2006), this was later realized to be an allele of *OsKSL8* (Toyomasu et al., 2016). Both alleles act upon *syn*-CPP, but that originally found in ssp. japonica (OsKSL8j) produces *syn*-stemarene (Nemoto et al., 2004), while that from ssp. indica (OsK-SL8i) produces *syn*-stemodene (Morrone et al., 2006). Most recently, such functional allelic variation was reported for *OsKSL10*, which primarily acts upon *ent*-CPP (Morrone et al., 2011), but that originally reported from ssp. japonica (OsKSL10j) produces *ent*-sandaracopimaradiene (Otomo et al., 2004a), while that from the wild rice most closely related to ssp. indica (i.e., *O. nivara*) produced *ent*-miltiradiene (Toyomasu et al., 2016), although this allele (OsK-SL10i) is widely found in ssp. japonica as well as ssp. indica in *O. sativa* (Feng et al., 2024).

Intriguingly, recent investigation of the allelic distribution of *OsKSL8* revealed even more phylogenetic diversity, with that from a representative cultivar (cv.) of ssp. basmati (cv. ARC 10497) exhibiting a distinct sequence relative to either of the previously characterized alleles (Zhao et al., 2023). Strikingly, it was more recently reported that this corresponds to a cross between *OsKSL8* and the neighboring pseudogene *OsKSL9p*, then termed *OsKSLX*, with the characterized allele (OsKSL-X-OL, here KSLXo) found to also act upon *syn*-CPP and produce *syn*-a-bieta-7,12-diene (**1**), the precursor of the phytoalexin oryzalactone (Kariya et al., 2024). This study also reported that *KSLX* was allelomorphic, with the presence of a functionally distinct allele that also acts upon *syn*-CPP but whose product was not identified (KSLX-NOL, here KSLXn), as well as a phylogenetically distinct allele associated with the African wild rice species *O. barthii* (KSLX-bar, here KSLXb), which was not further investigated (Kariya et al., 2024). Here is reported functional characterization of these two phylogenetically distinct alleles, both of which specifically act upon *syn*-CPP to produce *syn*-pimara-9(11), 15-diene (**2**), which has not previously been reported in rice, along with evolutionary analysis of the complex *KSL8/9/X* locus.

## 2. Results and discussion

**Fig. 1.**
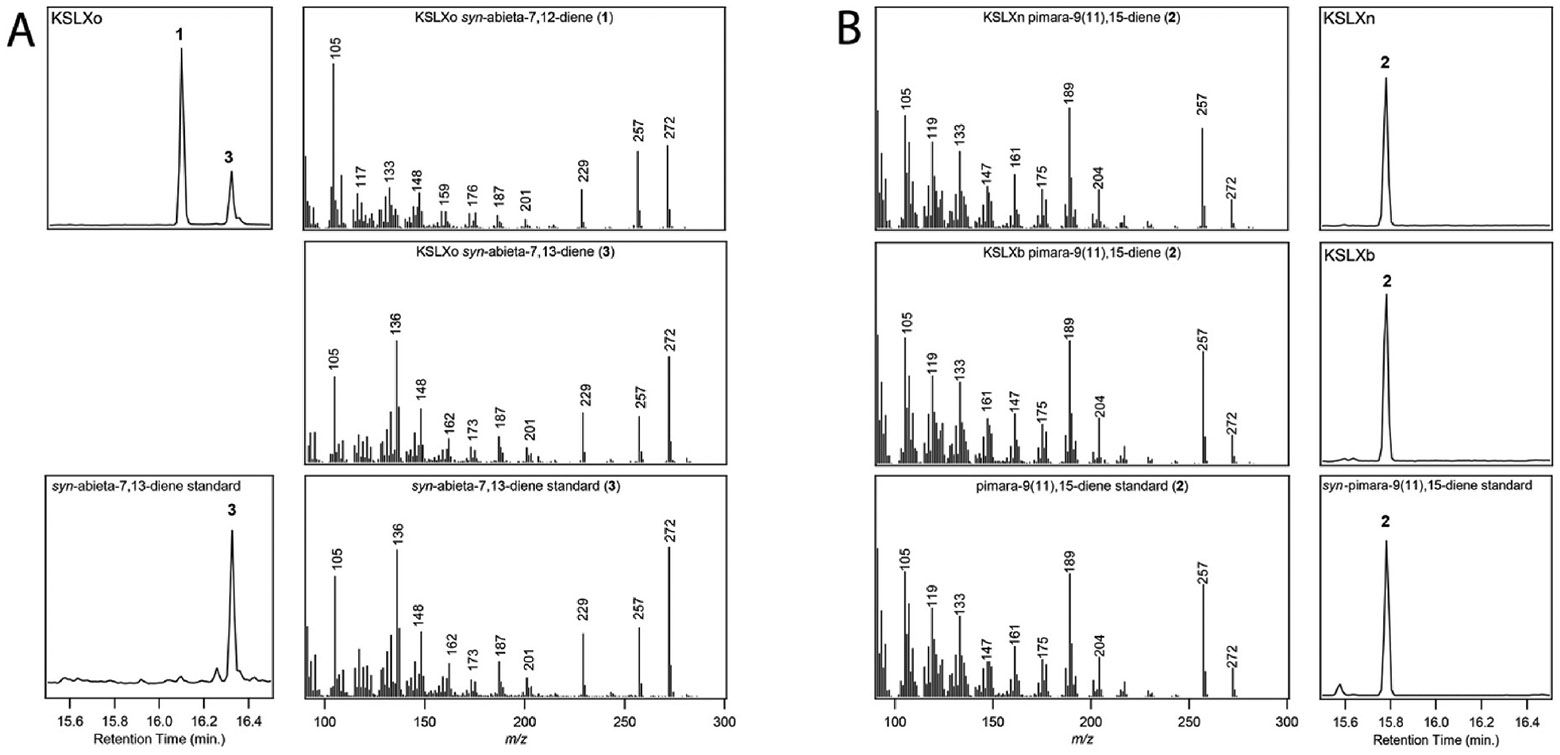
Identification of *KSLX* products from *syn*-CPP. GC-MS total ion chromatograms and mass spectra. A) For OsKSLXo, with authentic standard for minor *syn*-abieta-7,13-diene product (bottom). B) For OsKSLXn (top) and ObKSLXb (middle), with authentic standard for *syn*-pimara-9(11),15-diene (bottom).

To investigate the potential functional variation in the previously identified phylogenetically distinct allele from the ssp. basmati cv. ARC 10497 (Zhao et al., 2023), the pseudo-mature (i.e., without the N-terminal plastid targeting peptide) coding sequence was cloned and expressed in an *Escherichia coli* based modular metabolic engineering system (Cyr et al., 2007). The resulting construct was thus co-expressed with a GGPP synthase and CPSs producing either normal, *ent*- or *syn*-CPP, revealing this only acts upon *syn*-CPP. Given the 99.9 % amino acid (aa) sequence identity with the previously reported OsKSLXo (Kariya et al., 2024), as well as analogous mass spectra of both products (Supplemental Fig. S1), the major product was assumed to be **1** (Fig. 1A). In addition, the previously unidentified minor product was identified as the olefinic isomer *syn*-abieta-7,13-diene (**3**) based on comparison to a previously characterized enzyme (Chen et al., 2024; McCadden et al., in press).

Upon realization that KSLX was allelomorphic (Kariya et al., 2024), the previously reported OsKSLXn was obtained by gene synthesis. This was similarly truncated and examined *ex vivo* (i.e., via the metabolic engineering system) and found to also selectively act upon *syn*-CPP. By comparison to that previously reported from a bacterial class I diterpene synthase (Jia et al., 2019), the OsKSLXn product was identified as **2** (Fig. 1B).

The previously reported KSLXb revealed further phylogenetic diversity for this locus (Kariya et al., 2024). To investigate this, KSLXo was BLASTed against the genome of a representative landrace for *O. barthii* (Stein et al., 2018), which is readily accessible in the Gramene Oryza database (Tello-Ruiz et al., 2022). The best hit (Obart_032089, 89.2 % aa sequence identity) was similarly examined. This was synthesized with codon optimization for expression in *E. coli* as a pseudo-mature construct, which was then subjected to *ex vivo* analysis, demonstrating this ObKSLXb also selectively acts upon *syn*-CPP to produce **2** (Fig. 1B).

Intriguingly, closer examination of the *O. barthii* BLAST results revealed a significant difference for the larger *KSL8/9/X* locus. In particular, while it was previously reported that this locus consists either of *KSL8* and the adjacent pseudogene *KSL9p* (*KSL8-KSL9p*) or just the fused *KSLX* in *O. sativa* (Kariya et al., 2024), *O. barthii* contains only *KSL8* and *KSLXb*. This contrast implies *KSLXb* might correspond to a functional *KSL9*, the evolution of which is not well accounted for in the previously reported evolutionary analysis. Indeed, although strong phylogenetic evidence was presented for fusion of *KSL8* with *KSL9p* (via loss of the intervening sequence) giving rise to *KSLXo* and *KSLXn*, this was less clear for *KSLXb*. In particular, the 5′ region derived from *KSL8* in *KSLXo* and *KSLXn*, in *KSLXb* instead grouped with *KSL9p*, but was suggested to arise from an ancestor of *KSLX* rather than *KSL9*, leaving no functional version of the latter evident. While the larger 3’ region in *KSLX* forms a separate clade (including *KSLXb*) from *KSL9p*, this may reflect the loss of function and accompanying loss of selective pressure (Kariya et al., 2024). Here, it is hypothesized that *KSLXb* represents a functional copy of *KSL9*, providing a novel example of such retention. Regardless, given the bifunctional (CPS-KS) tridomain origin of the KSLs (Peters, 2025), the C-terminal domain containing the active site in KSLX is obviously derived from KSL9, with the evolution of distinct activity relative to *KSL8* providing enough of a selective advantage to enable an initial sweep of the tandem *KSL8-KSL9* pair into the population.

**Fig. 2.**
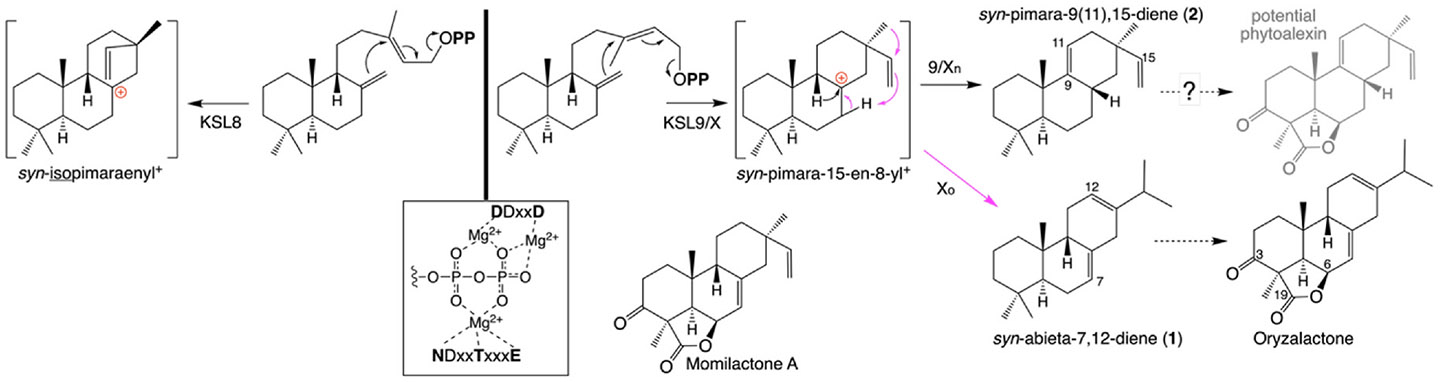
Scheme indicating reaction catalyzed by KSL9/X, along with contrasting initial cyclization catalyzed by KSL8 (left) and (boxed) conserved motifs ligating requisite trio of divalent magnesium ion co-factors (Mg^2+^) interacting with the pyro-phosphate (O**PP**) moiety, as well as *syn*-pimara-7,15-diene derived momilactone A and the oryzalactone derived from the KSLXo product *syn*-abieta-7,12-diene (**1**), with potential analogous derivative of the KSL9/Xn product *syn*-pimara-9(11),15-diene (**2**).

Although the reaction catalyzed by KSLXo leaves opaque the configuration at carbon-13 (C13) of the initially cyclized pimar-15-en-8-yl carbocation intermediate, it seems likely this corresponds to that observed in the OsKSLXn (and ObKSL9/Xb) product (i.e., α-methyl/β-vinyl). Notably, this differs from the C13-epimer (i.e., α-vinyl/β-methyl) *syn*-isopimaraenyl ^+^ intermediate necessary for the production of the further cyclized and rearranged *syn*-stemarene and *syn*-stemodene observed with OsKSL8 (Morrone et al., 2006). Accordingly, the gene duplication and neo-functionalization leading to KSL8 and KSL9/X led to divergence in the pre-catalytic conformation of the substrate to enable initial cyclization to form distinct C13-epimers of the resulting *syn*-pimar-15-en-8-yl^+^ intermediate (Fig. 2). Given the invariable position of the allylic pyrophosphate moiety whose lysis initiates the reaction, as dictated by the conserved aspartate-rich motifs required for binding the trio of divalent magnesium ion co-factors (Aaron and Christianson, 2010), this shift requires substantial remodeling of the active site. Indeed, the KSL9/Xs reported here share <73 % aa sequence identity with any of the KSL8 from the AA genome species available in the Gramene Oryza database, which is significantly less than the >85 % aa sequence identity found between the pairs in the two other tandem pairs of *KSLs* (i.e., KSL5/6 and KSL10/14).

**Fig. 3.**
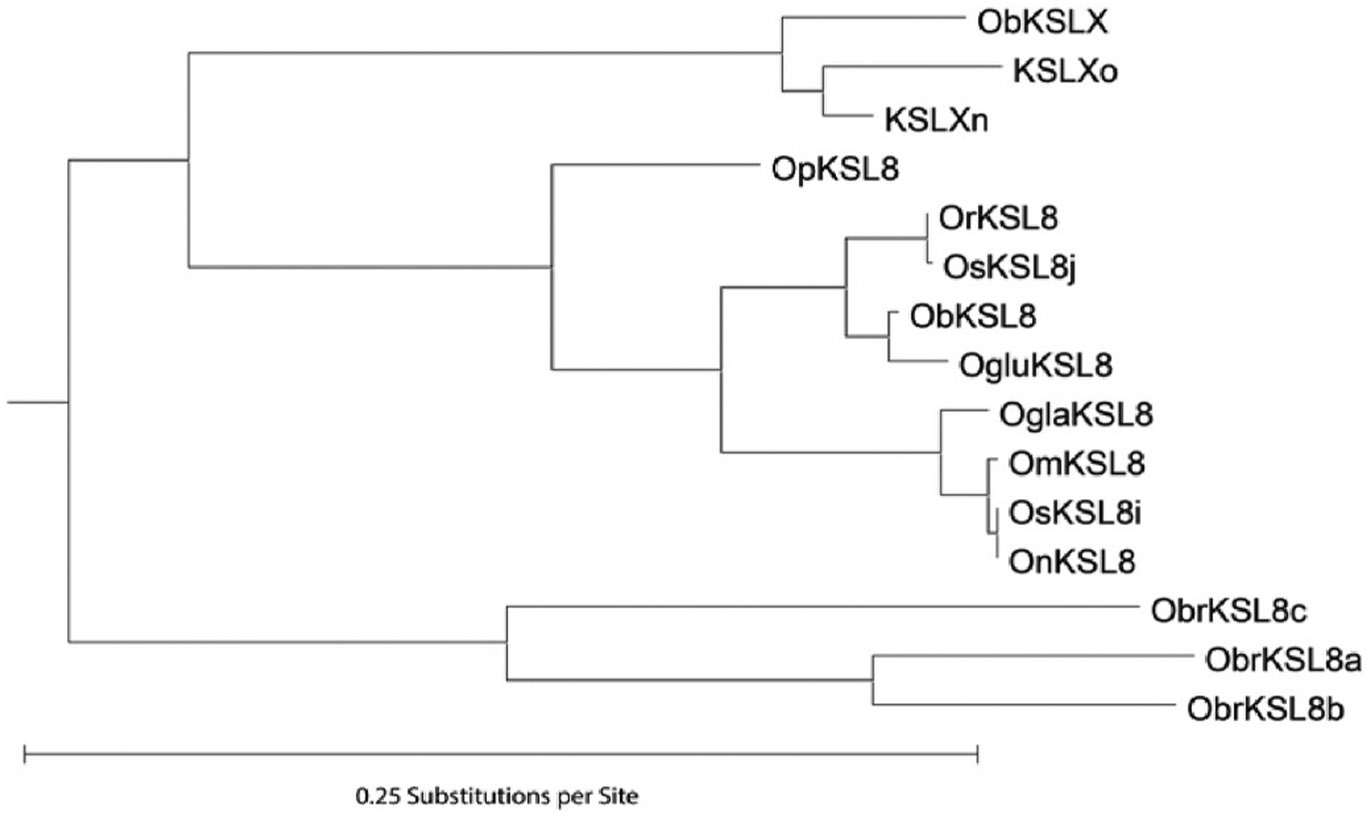
Phylogenetic tree for KSL8 and KSL9/X. Relevant clade extracted from broader *Oryza* KSL tree (Supplemental Fig. S2). The various species are indicated as follows: Ob, *O. barthii*; Obr, *O. brachyantha*, with its KSL8 paralogs designated a-c as described (Toyomasu et al., 2018); Oglu, *Oryza glumaepatula*; Ogla, *Oryza glaberrima*; Om, *Oryza meridionalis*; On, *O. nivara*; Op, *Oryza punctata*; Or, *O. rufipogon*; Os, *O. sativa*, with the distinct alleles of OsKSL8 associated with ssp. japonica or ssp. indica (as described in the text) designated as OsKSL8j and OsKSL8i (respectively).

Given such relatively large divergence, broader phylogenetic analysis was carried out to verify that KSL8/9/X form a clade within the KSL family from the species available in the Gramene Oryza database (Fig. 3 and Supplemental Fig. S2). Indeed, despite the observed divergence in sequence and function, KSL8 and KSL9/X group together and, hence, seem to have arisen from tandem gene duplication. It was previously reported that this duplication is specific to the AA and BB genome lineage, as the BB genome species *Oryza punctata* contains a homolog to *KSL8* (but not *KSL9/X*), while a homolog from the more distant (FF genome) species *Oryza brachyantha* was placed as an outgroup (Kariya et al., 2024). However, *O. brachyantha* has been reported to contain at least two functionally distinct paralogs of KSL8, albeit these react with normal rather than *syn*- CPP (Toyomasu et al., 2018). These ObrKSL8s were examined here and, while in the same clade, found to group with each other separately from KSL8 and KSL9/X, indicating separate/independent duplication and neo-functionalization in the two lineages (i.e., AA/BB versus FF).

Finally, the prevalence of *KSLX* in *O. sativa* was further investigated. As only a single example is present in Gramene Oryza (1 of 20 cultivars), while in the previously reported investigation of the sequenced members of the World Rice Core (WRC) collection (Tanaka et al., 2020), only 8 of the 69 available cultivars were found to have *KSLX* (Kariya et al., 2024), it seemed prudent to search for additional instances. Given the phylogenetic divergence of this locus, such a search requires high-quality, independently assembled genomes. Fortuitously, the Rice Super Pan database provides such a resource (Shang et al., 2022), and was utilized here. While *KSLX* was found in only 15 from the 202 available *O. sativa* cultivars, addition of this larger sample enables calculation of ~8 % prevalence for *KSLX* versus the more common *KSL8-KSL9p* at this locus.

Intriguingly, this compiled dataset also enabled investigation of sub-species specificity, revealing an imbalance. Specifically, of the 24 *KSLX* containing cultivars of *O. sativa*, 12 of the 15 containing *KSLXo* are from ssp. japonica, with one from the related ssp. basmati, and one each in ssp. indica and the related ssp. aus, while all 9 containing *KSLXn* are from ssp. indica, consistent with the general division of such functionally distinct *KSL* alleles between the japonica and indica sub-species (e. g., here *KSLXo* ≈ *KSLXj* and *KSLXn* ≈ *KSLXi*). Notably, this further indicates higher prevalence of *KSLX* in ssp. japonica (~15 %) than ssp. indica (~5 %). Moreover, given the expected association between *KSLXo* and production of oryzalactone, as already observed in the relevant WRC cultivars [c.f. (Kariya et al., 2023, 2024), this further implies oryzalactone is more prevalent in ssp. japonica. By contrast, the unknown LRD(s) derived from the *syn*-pimara-9(11),15-diene product of KSL9/Xn is limited even in ssp. indica. Based on the C3-keto and 19, 6β-olide (lactone) groups observed in both the *syn*-abieta-7,12-diene derived oryzalactone and *syn*-pimara-7,15-diene derived momilactone A, it can be speculated that these may be formed with *syn*-pimara-9(11), 15-diene as well, with the resulting LRD serving as a potential phytoalexin (Fig. 2).

## 3. Conclusions

**Fig. 4.**
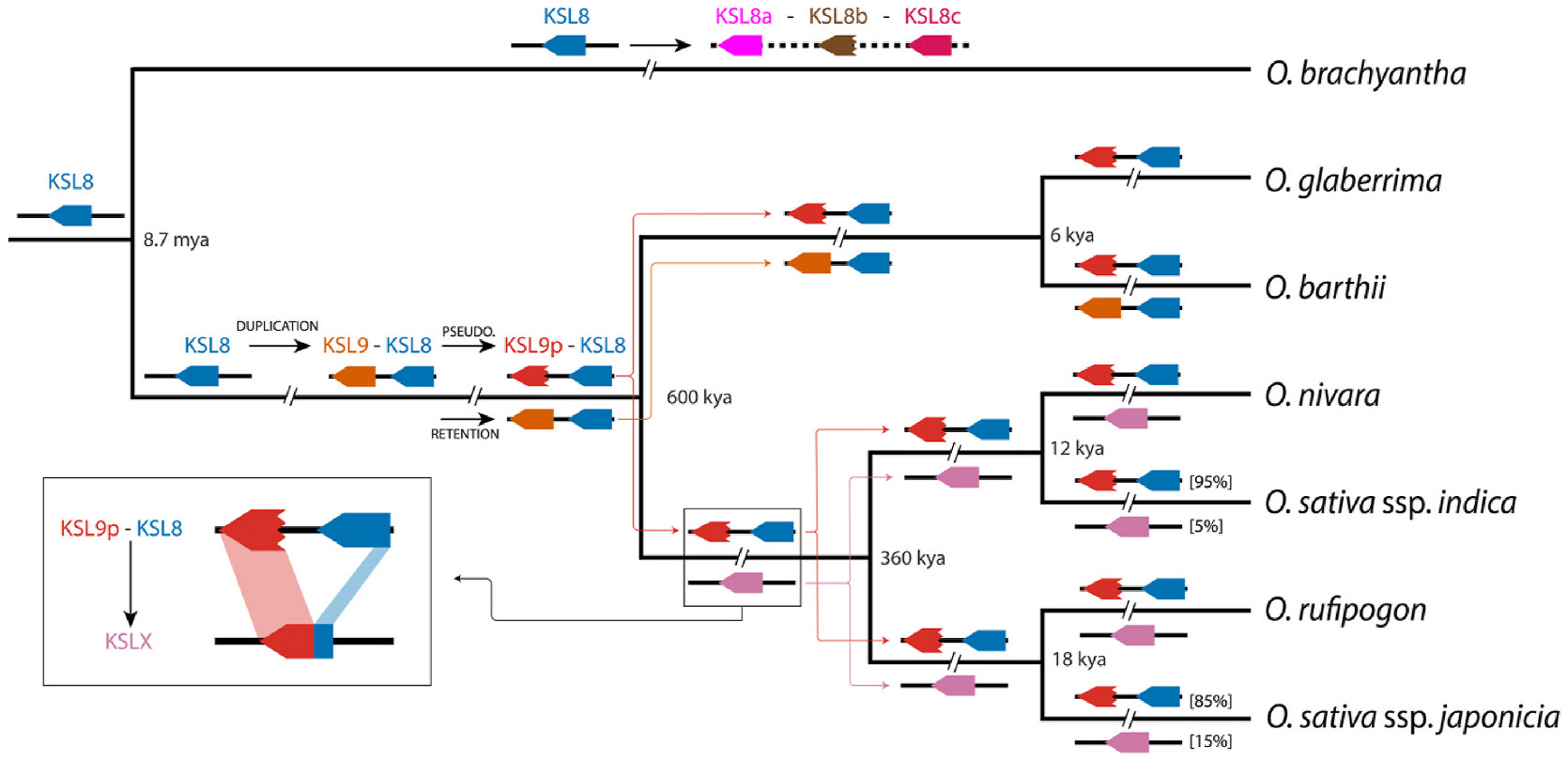
Hypothesized evolution of the *KSL8/9/X* locus in *Oryza*. Shown are genes at this locus in ancestral or extant species, with distinct composition indicated by stacking and, in the major japonica and indica sub-species of *O. sativa*, prevalence indicated in brackets. Note *O. brachyantha* is FF genome outgroup, which underwent its own independent gene duplication, while all other extant species shown here are from the AA genome group. Dates of divergence from (Wing et al., 2018).

The characterization of KSL9/X reported here imparts insight into evolution of the *KSL8/9/X* locus (Fig. 4). Assignment of *ObKSLXb* as *KSL9* indicates a functional copy of this gene was retained in the AA genome lineage, providing a rationale for the prevalence (>90 %) of *KSL9p* – i.e., the recent loss of function combined with selective pressure for retention of *KSL8*. Retention of the tandem *KSL8-KSL9p* pair then allowed crossing (presumably via homologous recombination) between *KSL8* and *KSL9p* to yield the functionally fused *KSLX* in the Asian rice lineage (i.e., *O. rufipogon/nivara/sativa*), which removes the need for the previously suggested separate derivation of *KSL9* and an ancestor to *KSLX* (Kariya et al., 2024). In addition, while this further implies less selective pressure for retention of *KSL9*, this is offset by the implied selective pressure leading to the almost certainly analogous activity of *KSLX*. Strikingly, the stronger prevalence of *KSLX* in ssp. japonica relative to ssp. indica presumably reflects the distinct activity exhibited by the associated KSLXo and, hence, resulting production of oryzalactone. Nonetheless, this still leaves a surprisingly high degree of complexity in the *KSL8/9/X* locus, with two distinct forms (*KSL8-KSL9p* and *KSLX*) that each further exhibit allelomorphism. Such complexity hints that this locus is under balancing selective pressures, presumably exerted by the competition phytopathogen variation exerts on phytoalexin biosynthesis, consistent with observations made with other loci associated with such defense (Ebert and Fields, 2020). Regardless, identification of this novel biosynthetic capacity provides further insight into the vast arsenal of LRD natural products encoded by the rice pan-genome.

## 4. Materials and methods

### 4.1. Biochemistry

Unless otherwise stated all chemicals were obtained from Fisher Scientific. *OsKSLXo* was cloned from ssp. basmati cv. ARC 10497 (OsARC_11g0013490) and truncated to remove the N-terminal plastid targeting sequence (first 68 aa). *OsKSLXn* (DDBJ Accession LC774657) was synthesized (Twist Biosciences) and truncated to remove the N-terminal plastid targeting sequence (again, the first 68 aa). *ObKSL9*/*Xb* (Obart_032089) was synthesized (Twist Biosciences) without the N-terminal plastid targeting sequence and optimized for expression in *E. coli* (see Supplemental data). The encoded enzymes were characterized using a previously described modular metabolic engineering system (Cyr et al., 2007). The resulting diterpene products were extracted, passed over silica gel to remove confounding polar metabolites and analyzed by gas chromatography with mass spectrometry (GC–MS) as previously described (Feng et al., 2024). *syn*-Pimara-9(11),15-diene was identified by matching retention time and mass spectra to the previously described product of the bacterial SaDTS with *syn*-CPP (Jia et al., 2019). *syn*-Abieta-7,13-diene was identified by matching retention time and mass spectra to the previously described product of the bifunctional bacterial StrDCS (Chen et al., 2024).

### 4.2. Bioinformatics

For construction of the *Oryza* KSL phylogenetic tree, KSLs from the *Oryza* species, with representative examples from both the japonica and indica sub-species of *O. sativa* (largely cv. Nipponbare and cv. IR8, respectively), available from the Grameme Oryza database were obtained by BLAST searches with the known examples for each, with the identified genes listed in Supplemental Table S1. In certain cases, particularly with KS1 and KSL2, the predicted mRNA for adjacent genes were fused. Alignment to the relevant KS(L)s from cv. Nipponbare was carried out to identify the amino acid sequence for each, enabling separation into distinct KS(L)s with the corresponding ranges indicated in Supplemental Table S1. Genes significantly shorter than the expected length of ~2400 AAs [or ~1700 AAs for KSL12, reported to lack the N-terminal γ-domain (Itoh et al., 2021)], were assumed to be pseudogenes and discarded. Pseudo-mature mRNA sequences of ObraKSL8a/c were obtained from GenBank accessions LC322116 and LC322118, which were then used in BLAST searches of Grameme Oryza to get the full-length predicted cDNA (Toyomasu et al., 2018). The obtained amino acid sequences were used for the presented phylogenetic analyses, which were carried out using RAxML-NG (Kozlov et al., 2019), with 50 random and 50 parsimony starting trees, followed by 1000 bootstrap replicates.

KSLX surveys were performed within Gramene Oryza, the World Rice Core Collection, and Rice Super Pan databases (Shang et al., 2022; Tanaka et al., 2020; Tello-Ruiz et al., 2022). The OsKSLXo and OsKSLXn reported here were used as BLAST queries in each database. Hits for all *Oryza sativa* cultivars were recorded and used to calculate the frequency of the genes within those populations (Supplemental Table S2).

## Supplementary Material

MMC1

Supplementary data to this article can be found online at https://doi.org/10.1016/j.phytochem.2025.114634.

## Data Availability

Data will be made available on request.
