# Supplementary material for: Uncrossing the ‘X’: Characterization of alternative alleles for KSLX in Oryza
Source: Phytochemistry. Author manuscript; Available in PMC 2025 Sep 2. (PMC12401587; doi:10.1016/j.phytochem.2025.114634)
Supplement: MMC1 [file NIHMS2104953-supplement-MMC1.pdf]

Supporting Information for:

Uncrossing the 'X': Characterization of alternative alleles for KSLX in *Oryza*

Tristan Weers, Yiling Feng & Reuben Peters*

Roy J. Carver Department of Biochemistry, Biophysics & Molecular Biology, Iowa State University, Ames, IA 50011, USA

* Corresponding author: e-mail address: rjpeters@iastate.edu

Table of Contents

Page 1: Sequence of synthetic ObKSL9/Xb (sObKSL9).

Page 2: **Fig. S1.** Comparison of mass spectra from OsKSLXo here and Kariya et al (2024).

Page 2: **Fig. S2.** Phylogenetic tree for *Oryza* KSLs.

Page 3: **Table S1.** Accession data for *Oryza KSL* family.

Pages 4-7: **Table S2.** Presence of *KSLX* in *O. sativa*.

>sObKSL9
ATGGTGAGGAAGCAGCTGCAGGGACTTGAGTTATCACCATCTTCATACGACACGGCATGGGTGGCCATGGTGCCAGTGCTGGGGTCTCCTCA
GTCTCCATGCTTCCCACGGTGTGTTGAGTGGATACTTCAGAATCAACAAGAGGACGGATCTTGGGGCCAGTCAGGGGTAGTCAACAAAGATG
CTCTCTTGTCTACCCTGGCATGTGTTCTTGCACTTAACACATGGAATGTTGGTCCAGATCACATCAGGAGAGGGCTTAATTTTATTGGAAGAAA
TTTCTCGGTTGCCATTGATGGGCAAAGTGTCGCTCCTGTGGGTTATTATATAAATTTTTCTGGTATGCTTAACCTTGCCATTGGGATGGGCTTG
GAAATCCCTGTCATGCAAACGGATATGAATGGTATTTTTTACCTTCGGGAGGTCGAATTGGAAAGGGATACTGGTGGCACAACTTTAGCGAG
GAAAGCCTTCATGGCGTATGTGTCAGAAGGGTTAGGGAAACTACAAGACTGGGATTATGTAATTGCATATCAAAGCAAGAACGGAACATTTT
TCAACTCACCTTCCACAACGGCCGCGGCAGCAATCTACAGTGGCAACGAAAGAGCACTAGACTCTTTGCGACTACTTACAACCAAATTGGGTG
GCCCAGTGCCAGCGATATATCCAGACAATATATACTCCCGGCTTTGCATGGTAGATACTCTAGAAAAGATGGGGATCTCTTTGAACTTTGCTT
GCGAGATACGAGATATATTGGACATGACTTATAGGTGTTGGATGCAAAATGAGGAGGAGATCATGTTGGATATGAAGCTGTGTGCAAAGGC
ATTTCGCCTCCTTCGTATGCACGGCTATAACGTCACCTCAGATGGGATGGCACAATTTGCTGAACAATCCAGCTTCGATAATTCACTTCATGCA
TATCTCAATGACATTGAGCCTTTGCTGGAGTTGTACAAAAGTTCACAAGTTCGCTTCTCGGAAGATGAGTTGATCCTACAAAATATTGGTTCCT
GGTCAACAAAATTACTCAAGAAACAACTCCCCTCCAAAAATATATCAAAATCTTTAAAGACAGAGGTTGAGTATGCCCTTAGATTCCCGCTCTA
CGCAACGCTGCAAACACAAGAGCACAGGAGGAACATTGAACGATTCAAGACAAATAGCATCCAGCTTCTAAAATCTGGACACTGCGGCTCCC
ACAAGAAGGAAGAAATTCTTGATTTGGCTATTCACGAATTCCATTCCACCCAGTCTATTTACCAACAAGAACTCCAGTATCTAAAGAGCTGGGT
AGCAGAATGTAGGCTGGAGGAGCTGGAATTCGCAAGGATAATGCCACTGCAGGCACTTTTGAGTGCTGTTCCTCCTCTGTTCCATCCTGAATT
ATCCGATGCCCGCATCGCATGGAGCCAGAACACTGTGCTGGTGACAGTGATGGATGACCTTTTCGATGGTGGAGGGTCCATGGAGGAGATG
AGAAACTTCGTCGCGCTAATCGAGAAATGGGACGAGCATAGTGAGATTGACATCTGCTCCAAGAACGTTGAGATTCTGTTCAATGCTGTTTAC
CACACGAACAAGCGCATTGGTGAAAAGGCCGCGCTGGTACAAAATCGCAGTGTCATGGATAATATTGCAGAGCATTGGCTGCTGATGGTGA
GGGCTATGATGACCGAGGCAGAGTGGGCGGCGAGCAAACGTATTCCGGCAACAATGGAGGAGTACATGTCGGCTGGGCGCCACTCCGTCG
TGGGCCCCATCATCCCGGTGGCGGCTTTCCTGGTGGGGCCGGAGCTGTCAGAGGAGGTTGTCAGGAGCGAGGAGTTCAGCCAGCTGATGAC
GCTCACGGCCACCATCAGCCGCCTCCTCAACGACATCATGACGTACGAGAGGGAGGTGGCTGCGGGGAAGCCCAACAGCGTCCTGCTACAT
GCCCTTGCCCTTGATGGTGGTGGTGGAGGAGGTGGCGGTAGTCCTTCTCCTGCAACGGTTGAAGCAGCCAAGGTGGAGATCGGGAGGACCA
TCCGGGAGTCCAGGTGGGACCTGCAGAGGCTGGTGTTCAGAGACGGCGGCATTGTTCCCCGACCAATCAGGGAGATGTTCTGGCAGACGAG
TAAGGTGGCCAGTGTCTTCTACCGGGACGGTGATCATTTCTCGCCCACGGAGATGCTCAGCGCTGTGAATGAGGTGATTATGGACCCACTCA
AAACTGCAAGCGAGGAACGCTACGAGTGA

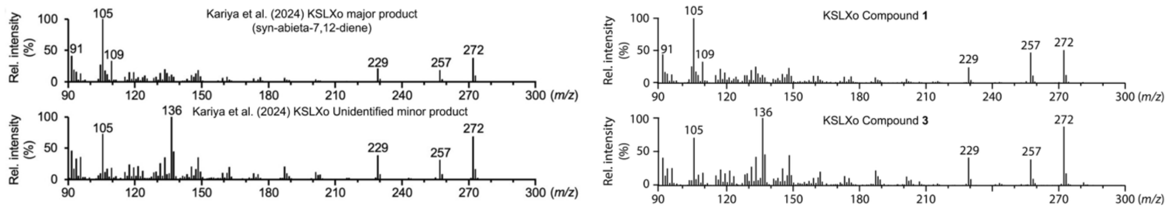

Fig. S1. Mass spectra of KSLXo major (**1**) and minor (**3**) products here (right) and from Kariya et al. (2024)(left).

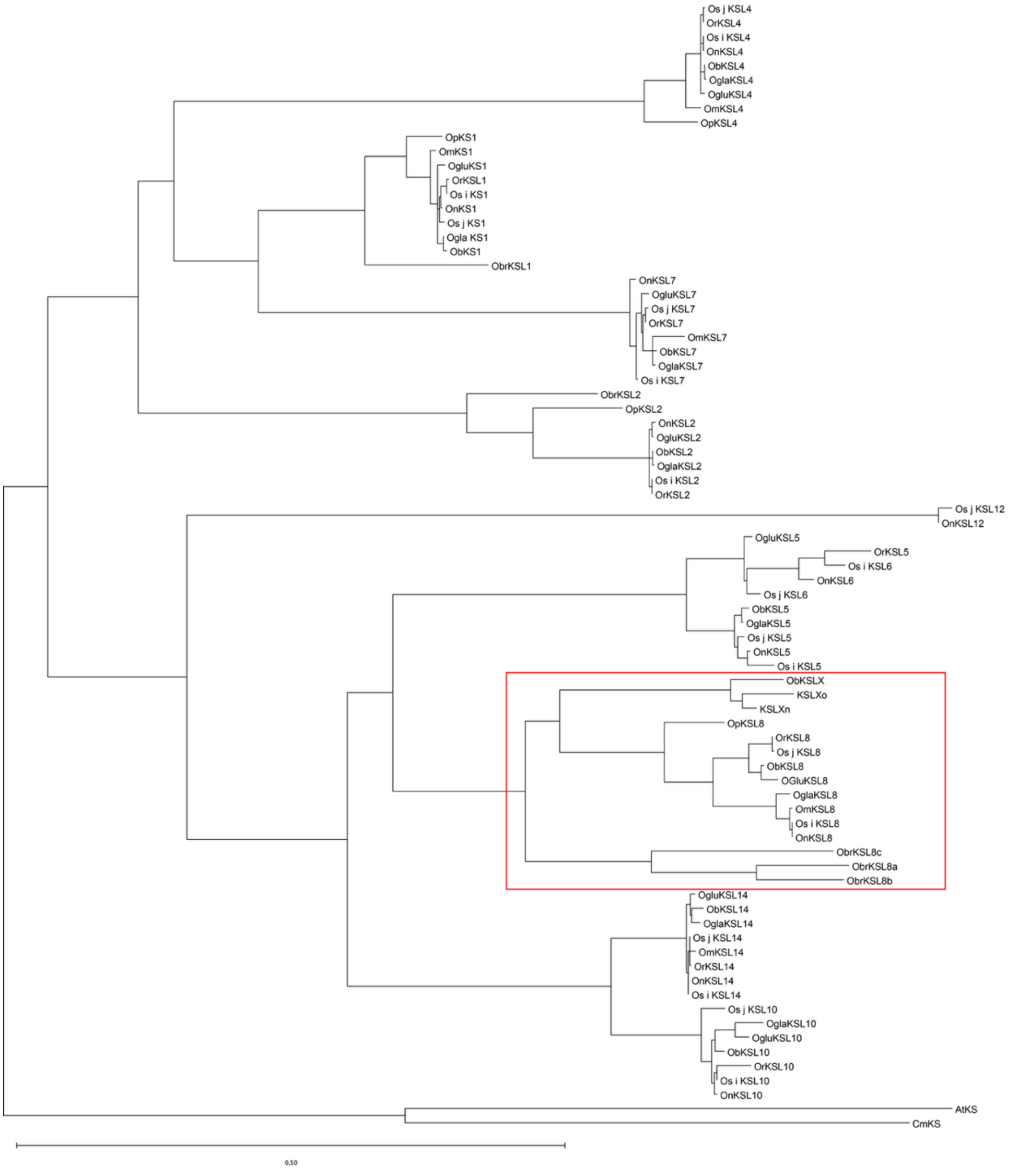

Fig. S2. Phylogenetic tree for *Oryza* KSLs. Red box is portion shown in Fig. 3, which indicates KSL8/9/X forms a complex locus, either *KSL8-KSL9(p)* tandem pair or *KSLX* (resulting from a cross between these). Accessions for each can be found in Table S1 (next page). KS from dicots *Arabidopsis thaliana* (AtKS) and *Cucurbita maxima* (CmKS) included as outgroup.

Species	KS1	KSL2	KSL4	KSL5	KSL6	KSL7	KSL8	KSL10	KSL12	KSL14
Sativa-japonica	Os04g0611800	Os04g0612000	Os04g0179700	Os02g0571300	Os02g0571800	Os02g0570400	Os11g0474800	Os12g0491800	Os02g0568700	OsKitaake12g133400
Sativa-indica	OsIR8_04G0250200: 1-756	OsIR8_04G0250200: 816-1558	OsIR8_04G0034900	OsIR8_02G02112[illegible]	OsGoSa_02g0022040	OsIR8_02G0210600	OsIR8_11G0130400	OsIR8_12G0130500	Not Found	OsIR8_12G0130300
Rufipogon	ORUFI04G26630:931-1641	ORUFI04G26630:1641-2424	ORUFI04G03600	ORUFI02G22440	ORUFI02G22410	ORUFI02G22350	ORUFI11G14170	ORUFI12G13740	Not Found	ORUFI12G13730
Nivara	Oniv_017679: 1-801	Oniv_017679: 801-1586	Oniv_015184	Oniv_012842	Oniv_012844	Oniv_012836	Oniv_036115	Oniv_012827	Oniv_012827	Oniv_039021
Barthii	Obart_017300:841-1564	Obart_017302	Obart_014815	Obart_012494	Obart_012498	Obart_012488	Obart_032092	Obart_034849	Not Found	Obart_034848
Glaberrima	Oglab_017288	Oglab_017289	Oglab_014781	Oglab_012461	Oglab_012464	Oglab_012456	Oglab_029228	Oglab_031996	Not Found	Oglab_031995
Glumaepatula	Oglum_017652: 1-803	Oglum_017652: 955-1695	Oglum_015170	Oglum_012833	Oglum_012832	Oglum_012827	Oglum_032687	Oglum_035453	Not Found	Oglum_035452
meridionalis	Omeri_017897	Omer_017901	Omeri_015298	Omeri_013010	Not Found	Omeri_013004	Omeri_033071	Omeri_035942	Not Found	Omeri_035941
punctata	Opunc_015115:1-747	Opunc_015115:748-1431	Opunc_013008	Not Found	Not Found	Not Found	Opunc_027923	Not Found	Not Found	Opunc_030239
brachyantha	Obrac_013386	Obrac_013387	Not Found	Not Found	Not Found	Not Found	*	Not Found	Not Found	Not Found

Pseudogene

Combined

8a	Obrac_027364
8b	Obrac_027367
8c	Obrac_027365

``

Database & Number	Name	Subspecies	KSLX Allele
Grameme	Chao Meo	Japonica	-
Grameme	Azucena	Japonica	-
Grameme	Ketan Nangka	Japonica	-
Grameme	Zhenshan 97	Indica	-
Grameme	IR64	Indica	-
Grameme	PR106	Indica	-
Grameme	Gobol Sail	Indica	-
Grameme	Larha Mugad	Indica	-
Grameme	Lima	Indica	-
Grameme	Khao Yai Guang	Indica	-
Grameme	Liu Xu	Indica	-
Grameme	Minghui 63	Indica	-
Grameme	N22	Aus	-
Grameme	Natel Boro	Aus	-
Grameme	ARC 10497	Basmati	-
Grameme	Nipponbare	Japonica	-
Grameme	93-11	Indica	-
Grameme	IR8	Indica	-
Grameme	Carolina	Japonica	-
Grameme	KitaakeX	Japonica	-
WRC02	Kasalath	Aus	-
WRC03	Bei Khe	Indica	-
WRC04	Jena 035	Aus	-
WRC05	Naba	Indica	-
WRC06	Puluik Arang	Indica	-
WRC07	Davao 1	Indica	-
WRC09	Ryou Suisan Koumai	Indica	-
WRC10	Shuusoushu	Indica	-
WRC11	Jinguoyin	Indica	-
WRC12	Da Hong Gu	Indica	-
WRC13	Asu	Indica	-
WRC14	IR 58	Indica	-
WRC15	Co 13	Indica	-
WRC16	Vary Futsi	Indica	-
WRC17	Keiboba	Indica	-
WRC18	Qingyu (Seiyu)	Indica	-
WRC19	Deng Pao Zhai	Indica	-
WRC20	Tadukan	Indica	KSLXn
WRC21	Shwe Nang Gyi	Indica	-
WRC22	Calotoc	Indica	KSLXn
WRC23	Lebed	Japonica	-
WRC24	Pinulupot 1	Indica	KSLXn
WRC25	Muha	Aus	-
WRC26	Jhona 2	Aus	-
WRC27	Nepal 8	Aus	-
WRC28	Jarjan	Aus	-
WRC29	Kalo Dhan	Aus	-
WRC30	Anjana Dhan	Aus	-
WRC31	Shoni	Aus	-
WRC32	Tupa 121	Aus	-
WRC33	Surjamukhi	Aus	-
WRC34	ARC 7291	Aus	-
WRC35	ARC 5955	Aus	-
WRC36	Ratul	Aus	-
WRC37	ARC 7047	Aus	-
WRC38	ARC 11094	Aus	-
WRC39	Badari Dhan	Aus	-
WRC40	Nepal 555	Aus	-
WRC41	Kaluheenati	Aus	-
WRC42	Local Basmati	Aus	KSLXo
WRC43	Dianyu 1	Japonica	-
WRC44	Basilanon	Indica	KSLXn
WRC45	Ma sho	Japonica	-
WRC46	Khao Nok	Japonica	-
WRC47	Jaguary	Japonica	KSLXo
WRC48	Khau Mac Kho	Japonica	-
WRC49	Padi Perak	Japonica	-
WRC50	Rexmont	Japonica	-
WRC51	Urasan 1	Japonica	-
WRC52	Khau Tan Chiem	Japonica	KSLXo
WRC53	Tima	Japonica	-
WRC55	Tupa729	Japonica	-
WRC57	Milyang 23	Indica	-
WRC58	Neang Menh	Indica	-

WRC59	Neang Phtong	Indica	-
WRC60	Hakphaynhay	Indica	-
WRC61	Radin Goi Sesat	Indica	-
WRC62	Kemasin	Indica	-
WRC63	Bleiyo	Indica	-
WRC64	Padi Kuning	Indica	-
WRC65	Rambhog	Indica	-
WRC66	Bingala	Indica	-
WRC67	Phulba	Japonica	-
WRC68	Khao Nam Jen	Japonica	KSLXo
WRC97	Chin Galay	Indica	-
WRC98	Deejiaohualuo	Indica	-
WRC99	Hong Cheuh Zai	Indica	-
WRC100	Vandaran	Indica	-
NH001	Heibiao	Japonica	-
NH002	Sansuijin	Japonica	-
NH003	Zaoshengbai	Japonica	-
NH005	Wanshi	Japonica	-
NH006	Yikong	Indica	-
NH007	Baxiang	Indica	-
NH008	Vietnam Zaodao	Indica	-
NH009	Malaihong	Indica	-
NH010	CO 22	Indica	-
NH011	2037(Rajahamsal)	Indica	-
NH012	Sri Lanka 1	Indica	-
NH013	Wuziluosi 215	Japonica	-
NH014	Kahamu	Indica	-
NH016	Aerjituo	Japonica	-
NH017	American Huangkedao	Japonica	-
NH018	Buleida A-75	Indica	-
NH019	Seln 244A6-20	Indica	-
NH021	Xianluosichi	Indica	-
NH022	Djanda Mandja	Japonica	-
NH024	C 894-21	Indica	-
NH025	IR 10179-23-1-3	Indica	-
NH026	Dumai	Aus	-
NH027	Jaibattey	Indica	-
NH028	Rohini	Indica	-
NH029	BW 293-2	Indica	-
NH030	Albania Rice	Japonica	-
NH033	Nanoay P.A	Japonica	-
NH034	Nabated A Smar	Japonica	KSLXo
NH035	IRAT 10	Indica	-
NH036	K 24	Indica	-
NH038	Qianchonglang2	Japonica	-
NH039	Gongchengxiang	Japonica	-
NH040	Qiutianxiaoting	Japonica	-
NH041	Zhenfu 8	Japonica	-
NH042	CISOKAN	Indica	-
NH044	GZ 1368-5-4	Indica	-
NH045	J34	Indica	-
NH046	80A97YR303-304-1-3	Japonica	-
NH047	80050YR72136-43	Japonica	-
NH048	YR196	Japonica	-
NH051	Nanjing 11	Indica	-
NH052	Aijiaonante	Indica	-
NH053	Guangluai 4	Indica	-
NH054	Nantehao	Indica	-
NH055	Guizhao 2	Indica	-
NH056	Xiangzaoxian 7	Indica	-
NH057	Huangsiguizhan	Indica	-
NH058	Funingzipigengzi	Indica	-
NH060	Dandongludao	Japonica	-
NH061	Laoguangtou 83	Japonica	-
NH062	Muxiqiu	Japonica	-
NH063	Qiuqianbai	Indica	-
NH064	Jinxibai2	Indica	KSLXn
NH065	Taishannuo	Indica	-
NH066	Jinbaoyin	Indica	-
NH067	Minbeiwanxian	Indica	-
NH069	Esiniu	Indica	-
NH070	Heidu 4	Indica	-
NH072	WYJ7	Japonica	-
NH073	Bawangbian 1	Indica	-
NH074	Xugunuo	Indica	-

NH076	San**Qishiluo	Indica	-
NH077	Qitoubaigu3	Indica	-
NH079	Xiaohonggu	Indica	-
NH080	Gongju 73	Indica	-
NH081	Qitougu	Indica	-
NH082	Zinuo	Indica	-
NH083	Mowangguneiza	Indica	-
NH084	Jinzhinuo4	Indica	-
NH085	Jiienuo	Indica	-
NH087	Yangkenuo	Japonica	-
NH089	Laohongdao	Japonica	KSLXo
NH090	Jiabala	Indica	-
NH091	Baoxuan 21	Indica	-
NH092	Wenxiangnuo	Indica	-
NH093	TQ	Indica	-
NH094	Xianggu	Indica	-
NH096	Zimangfeie	Japonica	-
NH097	Liusha 1	Indica	-
NH098	Chenwan 3	Indica	-
NH099	Chengduai 3	Indica	-
NH100	Aimakang	Indica	-
NH101	Shufeng 101	Indica	KSLXn
NH103	Guangluai 15-	Indica	-
NH104	Hongwan 1	Indica	-
NH105	Luke 3	Indica	-
NH107	Zaoshuxiangheimi	Indica	-
NH109	Xiangwanxian 3	Indica	-
NH110	Zaoxian 240	Indica	-
NH111	Dangyu 5	Indica	-
NH112	Hongainuo	Indica	-
NH113	Wanlixian	Indica	-
NH114	Aizizhan	Indica	-
NH115	Xiaobaimi	Indica	-
NH116	Yanshuichi	Indica	KSLXo
NH117	Xishi 15	Japonica	-
NH118	Honggenghangu3	Indica	-
NH119	Yuyannuo	Japonica	KSLXo
NH120	80B	Indica	-
NH122	IR 661-1	Indica	-
NH123	Pei C122	Indica	-
NH125	Ninghui 21	Japonica	-
NH128	Teqingxuanhui	Indica	-
NH129	JWR 221	Indica	-
NH130	Baikehanhe	Indica	-
NH131	Lengshuinuo	Japonica	KSLXo
NH132	Haoxiang	Indica	-
NH133	L 301B	Indica	-
NH134	Jinnante 43B	Indica	-
NH135	Zaoshunonghu 6	Japonica	-
NH136	Qingsiai 16B	Indica	-
NH137	Liming B	Japonica	-
NH138	Baoxie -7B	Indica	-
NH139	G Zhenshan 97B	Indica	-
NH140	Taizhongzailai 1/taizhong65	Indica	-
NH141	Yelicanghua	Japonica	-
NH142	Baigedao	Japonica	-
NH143	Liushizao	Indica	-
NH144	Qingke	Aus	-
NH145	Jianghuadao	Japonica	-
NH146	Zhuyuan	Japonica	-
NH147	Ailuyu	Japonica	-
NH148	Hongse 90	Japonica	-
NH149	Linguo	Japonica	-
NH150	Tjantajan	Indica	-
NH151	BRC 25-146-2-1	Indica	-
NH152	Keluoduo B	Japonica	KSLXo
NH153	P1790-5-1M-4-5M-1B-3M-B	Indica	KSLXn
NH155	Chimao	Japonica	-
NH156	Qingnuo Kyohatamochi	Japonica	-
NH157	CHANH 148	Indica	KSLXn
NH158	SLK 2-18-2	Indica	-
NH159	RP 1570-44-1	Indica	-
NH166	ECIA 179-S13	Indica	-
NH167	PMS 10B	Indica	-
NH168	Taidongludao	Japonica	-

NH170	Jiefangxian	Indica	-
NH171	Hongmisandan	Japonica	-
NH173	Chengnongshuijing	Indica	-
NH174	Biwusheng	Indica	-
NH175	Longhuamaohu	Japonica	KSLXo
NH176	Cunsanli	Japonica	KSLXo
NH177	Aihechi	Indica	-
NH178	Lucaihao	Indica	-
NH179	Nanxiongzaoyouzhan	Indica	-
NH181	NJ6	Indica	-
NH182	Xuanenchangtanqingzhan	Indica	-
NH183	Hanmadao4	Indica	-
NH184	Honggu	Indica	-
NH186	IR24	Indica	-
NH187	Fanhaopi	Indica	-
NH189	Cungunuo	Japonica	KSLXo
NH190	Younian	Indica	-
NH191	Heimangdao	Japonica	-
NH192	Menjiagao 1	Indica	-
NH193	Haobayong 1	Japonica	-
NH194	Menjiading 2	Indica	-
NH196	Dongtingwanxian	Indica	-
NH197	Xiangaizao 10hao	Indica	-
NH198	Xiangwanxian 1	Indica	-
NH199	Aituogu 151	Indica	-
NH201	Jindao 1	Japonica	-
NH202	Momi	Indica	-
NH203	Zhendao 232	Indica	-
NH204	Zhengdao 5	Japonica	KSLXo
NH205	Lamujia	Japonica	-
NH206	Huhui 91269	Indica	-
NH207	Xiangdao	Indica	-
NH208	Laozaogu	Indica	-
NH210	Zhuzhen B	Indica	-
NH211	Chaoyangyihao B	Indica	-
NH212	lemont	Japonica	-
NH213	Xiangai B	Indica	-
NH214	Jiangnongzao 1 B	Indica	-
NH215	Jinghu3 B	Indica	-
NH216	Dianrui 409B	Indica	-
NH218	RD23	Indica	-
NH219	Mamagu	Indica	-
NH220	Meihuanuo	Indica	-
NH221	Weiguo	Japonica	-
NH222	Sanbaili	Indica	-
NH223	Haomake (K)	Japonica	-
NH224	Nangaogu	Indica	-
NH232	RY2	Japonica	-
NH237	Miyang46	Indica	-
NH238	WYJ3	Japonica	-
NH239	IR26	Indica	-
NH240	LanSheng	Japonica	-
NH242	ZCD	Japonica	-
NH243	Tetep(a)	Indica	KSLXn
NH246	GM4	Indica	-
NH247	DGB	Indica	-
